# Social Eavesdropping in Zebrafish: Tuning of Attention to Social Interactions

**DOI:** 10.1038/srep12678

**Published:** 2015-08-05

**Authors:** Rodrigo Abril-de-Abreu, José Cruz, Rui F. Oliveira

**Affiliations:** 1 Instituto Gulbenkian de Ciência, Rua da Quinta Grande 6, 2780-156, Oeiras, Portugal; 2 ISPA - Instituto Universitário, Rua Jardim do Tabaco 34, 1149-041 Lisboa, Portugal; 3 Champalimaud Neuroscience Programme, Champalimaud Centre for the Unknown, Av. de Brasilia, 1400-038 Lisboa, Portugal

## Abstract

Group living animals may eavesdrop on signalling interactions between conspecifics in order to collect adaptively relevant information obtained from others, without incurring in the costs of first-hand information acquisition. This ability (aka social eavesdropping) is expected to impact Darwinian fitness, and hence predicts the evolution of cognitive processes that enable social animals to use public information available in the environment. These adaptive specializations in cognition may have evolved both at the level of learning and memory mechanisms, and at the level of input mechanisms, such as attention, which select the information that is available for learning. Here we used zebrafish to test if attention in a social species is tuned to the exchange of information between conspecifics. Our results show that zebrafish are more attentive towards interacting (i.e. fighting) than towards non-interacting pairs of conspecifics, with the exposure to fighting not increasing activity or stress levels. Moreover, using video playbacks to manipulate form features of the fighting fish, we show that during the assessment phase of the fight, bystanders’ attention is more driven by form features of the interacting opponents; whereas during the post-resolution phase, it is driven by biological movement features of the dominant fish chasing the subordinate fish.

Public information, a form of inadvert social information^1^, is widely available at low cost to animals living in social groups, who can use it to adjust their behaviour to a dynamic social environment. For example, animals may eavesdrop on signalling interactions between third parties to collect information on the relative competitive ability of the opponents, without incurring in the costs associated with fighting and which they may use on subsequent interactions with the observed individuals^2^. This ability to collect and use adaptively relevant information from others (aka social eavesdropping)^3^,^4^ may thus impact the Darwinian fitness of the animal. Therefore, it has been proposed that group living has led to selection for the evolution of cognitive processes that enable animals to take advantage of the public information available in the social environment^5^,^6^,^7^. Some authors have suggested that these cognitive adaptations for social living depend on a set of domain-specific modules that evolved specifically for this purpose, and consequently the mechanisms involved in social learning would differ from those of individual learning^8^. However, this hypothesis has been recently challenged by accumulating evidence which suggests that: (1) both social and individual (asocial) learning share general associative learning mechanisms^9^; (2) social and asocial learning abilities co-vary across and within species (i.e. the better an animal performs in social learning tasks, the better it also performs in asocial learning tasks^10^,^11^,^12^); and (3) even solitary species can exhibit social learning^13^,^14^. Together these results have questioned the evolution of social learning as an adaptive specialization for group living and suggest that social and asocial learning share the same underlying mechanisms^9^,^15^.

As a consequence, it has recently been proposed that adaptive specializations in social cognition may have evolved at the level of input mechanisms, such as perception, attention, or motivation, which select the information that becomes available for learning, rather than at the level of learning mechanisms^9^. Despite the extensive literature on the adaptive function of social learning, research on its neural and cognitive mechanisms has been more scarce^16^, and this “black-boxing” of mechanisms may limit our understanding of its functional role. From the four basic cognitive processes involved in learning – acquisition, encoding, storage and retrieval of information - the former is related to the input mechanisms that select information available for learning, and the latter three are related to the long-term encoding of relevant information. Therefore, social and asocial information may share similar encoding, storage and retrieval mechanisms, but social species may be more tuned to social information available in the environment.

Input mechanisms, such as perception, attention and motivation, are crucial for higher-level cognitive processes, since they determine which information is selected for subsequent processing. Indeed, each species sensory specializations define a species-specific perceptual space, i.e. the *umwelt* or “self-world” as proposed by Jakob von Uexküll (1934)^17^, which allows an individual to respond adaptively to their environment in terms of appropriate responses to its own food, mates, competitors and predators. Subsequently, sensory information is filtered by perception and attention, providing information that becomes available for learning and decision-making processes. The relevance of attentional processes for learning has been recently highlighted; in particular, individuals’ selective attention efficacy has been shown to co-vary with performance on a wide range of cognitive tasks, which is taken as measure of a general intelligence trait^18^. Finally, motivation also plays a key role in the input mechanisms since it can direct attention to relevant stimuli in the environment by enhancing their salience, as exemplified by fear enhanced or hunger reduced vigilance towards predators in foraging fish^19^,^20^. In summary, adaptive specializations in input mechanisms can contribute as much as those in higher-level cognitive processes for the evolution of adaptive behaviours. Given that conspecifics are a significant component of the environment, it is expected that adaptive specializations have evolved to tune these input mechanisms towards relevant social information, in particular, to intercept the exchange of information between conspecifics.

Here we investigate if individuals of a highly social species, the zebrafish (*Danio rerio*), are tuned to attend to social information exchanged between conspecifics. The zebrafish has been emerging as a model organism in behavioural neuroscience and is a promising model for the study of the proximate mechanisms underlying social cognition^21^,^22^,^23^. On one hand it is highly social (i.e. expresses a strong preference to associate with conspecifics^24^,^25^,^26^), lives in mixed-sex groups (shoals) with structured dominance hierarchies^27^,^28^, is able to discriminate familiar from unfamiliar individuals^29^,^30^, and exhibits behavioural flexibility dependent on social experience (e.g. winner and loser effects^31^); moreover, attention paradigms have already been successfully used in zebrafish^32^,^33^. On the other hand, there is an extensive genetic toolbox available for this species (e.g. sequenced and annotated genome^34^; transgenic lines for visualization and manipulation of neural circuits^35^,^36^), which allows both the search for candidate genes and gene networks, as well as unveiling the neural circuits underlying relevant cognitive traits.

In order to test if zebrafish males pay attention to social interactions between other conspecific males, we used a one-trial preference task, where a bystander male zebrafish could observe without being observed: (1) an agonistic interaction (fight) between two male conspecifics; (2) two non-interacting male conspecifics; or (3) an empty tank (reference treatment). Bystanders’ behaviour was used as a read out of attention by using a combination of measures, such as sustained proximity, body orientation and directional focus. We predict that bystander zebrafish will pay more attention to conspecifics when they are engaged in social interactions, and therefore exchanging social information, than when conspecifics are present in the environment but not interacting. Next we used video playbacks to manipulate social features present in the interaction in order to identify key features that drive their attention. Specifically we compared the attentional response to a video recorded fight interaction, where the interacting fishes’ features were edited, such that the pattern of movement remained the same but body features were absent. This was achieved by replacing the fish on each frame by dots with the same surface area and mean colour (i.e. fighting fish vs. fighting dots).

## Results

### Bystander zebrafish pay attention to social interactions

**Figure 1:**
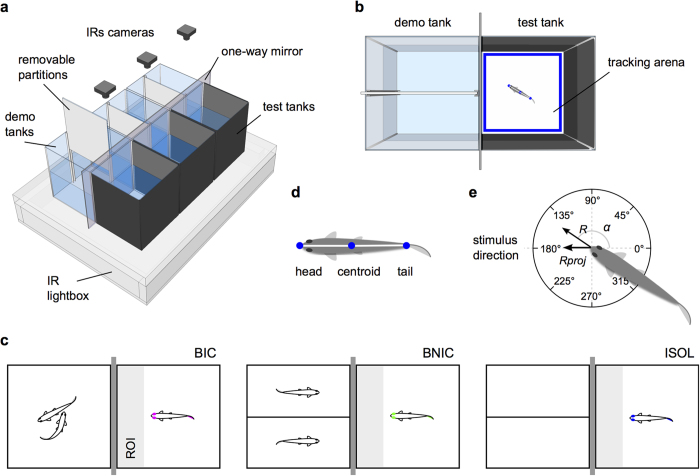
Behavioural paradigm. (**a**) 3D diagram of the experimental setup. Fixed IRs cameras record all behavioural tests from above (see Methods). (**b**) Top view diagram of a demonstrator + test tank with focal fish. Each tracking arena (blue rectangle) is defined post-test for offline tracking of the recorded videos. (**c**) Schematic of the experimental treatments: bystander to fighting conspecifics (BIC); bystander to non-interacting conspecifics (BNIC); and socially isolated (ISOL). Focal fish represented with colour (BIC- magenta; BNIC- lime; ISOL- blue) and demonstrator fish (stimuli) represented in black. Region of interest (ROI) represented in light grey and one-way mirror in dark grey. (**d**) Schematic of the focal fish’s tracking points (blue dots) used for coordinates extraction. (**e**) Schematic of the focal fish’s possible mean orientations measured by its centroid-to-head axis angle *α*. 0° opposite and 180° directed towards the stimulus tank direction. *R* represents the mean resultant vector’s length and *R proj* its projection onto the stimulus direction.

**Figure 2:**
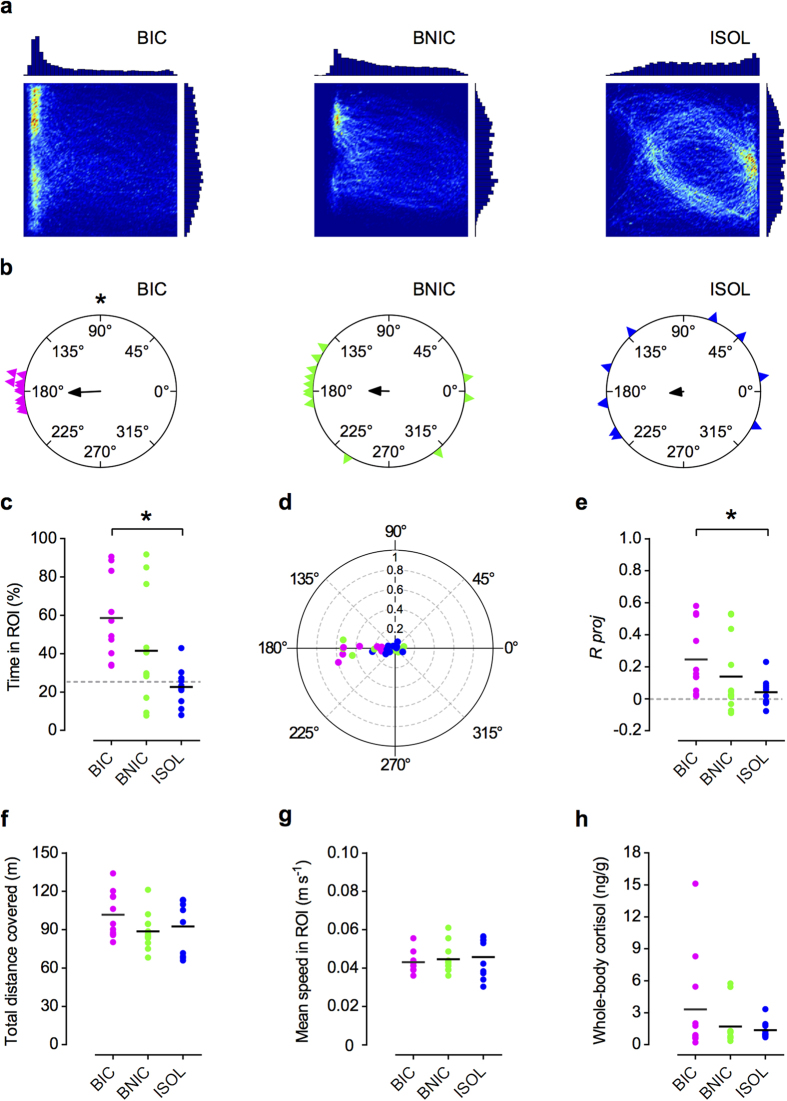
Bystanders’ behavioural and hormonal results. (**a**) 2D heatmaps and linear histograms of the time spent in each position of the tracking arena by a representative focal fish from each treatment: bystander to fighting conspecifics (BIC); bystander to non-interacting conspecifics (BNIC); and socially isolated (ISOL). The heatmaps are scaled from maximum relative value (red) to minimum relative value (dark blue). Linear histograms represented in arbitrary scale. (**b**) Circular plots of the focal fishes’ individual mean orientations for each treatment (BIC – magenta triangles, BNIC – lime triangles, ISOL – blue triangles) and the corresponding group mean resultant vector (black arrows). BIC fish deviate significantly from a uniform distribution, clustering around its group mean resultant vector. (**c**) Scatter plot (n = 10 to 12 / treatment) of the individual (coloured dots) and mean (black lines) percentage of time spent in the ROI for each treatment (BIC– magenta; BNIC– lime; ISOL– blue) during the 30 minutes test. Dashed grey line represents the value expected from a random distribution in the arena (25%). (**d**) Polar scatter plot of the focal fishes’ (coloured dots) individual mean resultant vector’s angles *α* (0˚ to 360˚) combined with corresponding vector lengths *R* (0 to 1), for each treatment. (**e**) Scatter plot of the individual (coloured dots) resultant vector’s lengths *R* projected (*R proj*) onto the stimulus direction (180˚) and corresponding group mean value *R*_*g*_ *proj* (black lines), for each treatment. Positive values indicate directional focus towards the stimulus; zero indicates no directionality (dashed grey line); negative values indicate directional focus opposite to the stimulus. (**f,g**,**h**) Scatter plots of the individual (coloured dots) and mean values (black lines) of the focal fishes’ total distance covered in the arena, mean speed in ROI and whole-body cortisol levels, for each treatment. * *P* < 0.05, ** *P* < 0.01, *** *P* < 0.001.

**Figure 3:**
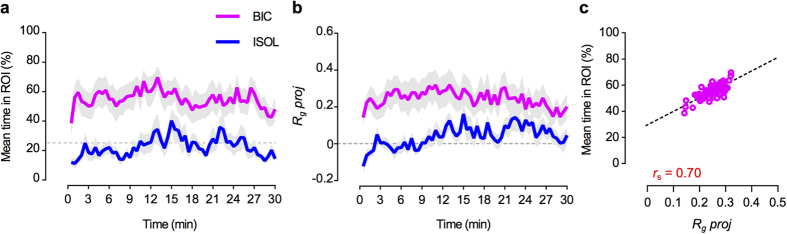
Bystanders’ temporal dynamics of proximity and directional focus towards fighting conspecifics. (**a**) Comparison between the bystanders to fighting conspecifics’ (BIC) mean time in the ROI and the socially isolated (ISOL) reference fish, measured in 30 seconds bins, throughout the 30 minutes test. (**b**) Comparison between the BIC and ISOL fishes’ mean directional focus onto the stimulus direction (*R*_*g*_ *proj)*, measured in 30 seconds bins, throughout the 30 minutes test. For both (**a**) and (**b**), the coloured thick lines (BIC- magenta; ISOL- blue) represent the mean values for each treatment. Grey shadows represent the standard error (SEM). The dashed grey line represents in (**a**) the value expected from a random distribution in the arena (25%); in (**b**) no directionality (*R*_*g*_ *proj* = 0). (**c**) Scatter plot of the BIC fishes’ mean time spent in the ROI as function of *R*_*g*_ *proj*. Open magenta circles represent the sampled (in 30 seconds bins) means. The Spearman correlation coefficient *r*_s_ is shown in red. Dashed line indicates the regression line for easier visualization of trend.

In order to test if zebrafish males pay particular attention to social interactions, we analysed for 30 minutes a set of behavioural parameters of individual bystander fish, placed in our experimental setup (see Methods and Fig. 1a,b), in one of three experimental treatments (Fig. 1c): (1) bystander to interacting (i.e. fighting) conspecifics (BIC); (2) bystander to non-interacting conspecifics (BNIC); and (3) socially isolated (ISOL). We video recorded all focal fishes’ behaviour and developed a custom-made video tracking system for offline tracking of each fish’s body position (head, centroid, tail; Fig. 1d) inside a defined region (arena) of the test tank (see Methods, Fig. 1b and Supplementary Video 1). Attentiveness of each focal fish was inferred from its position in the arena and from its body orientation (i.e. directionality; Fig. 1e) relative to the stimulus. Four behavioural parameters, one qualitative and three quantitative, were used as read-outs: (1) the spatial distribution of the focal fish in the arena (Fig. 1b); (2) time spent in the vicinity of the stimulus fish[i.e. time in a region of interest (ROI); Fig. 1c]; (3) orientation towards the stimulus fish (*α*, Figs 1e); and (4) directional focus towards the stimulus fish (*R proj*, Fig. 1e).

We started the analysis by individually profiling the time spent by the focal fish in each position of the arena during the test. Qualitative analysis using 2D heatmaps and histogram representation, revealed different spatial distribution patterns for each treatment (Fig. 2a). On average BIC fish spent more time closer to the tank wall on the side of the fighting stimulus fish than did BNIC fish, which showed a more dispersed distribution in the arena. A few BNIC fish spent more time in the area closer to one of the two non-interacting stimulus fish. ISOL fish showed on average a dispersed distribution in the arena. As a quantitative measure of proximity towards the stimuli, we analysed for each treatment the group mean percentage of time spent in the region of interest (ROI), comprising 25% of the tank (see Methods, Fig. 1c). BIC fish spent significantly more time in the ROI than ISOL fish, whereas there were no differences between BNIC and ISOL fish (see Fig. 2c and Table 1). The differences between BIC and BNIC fish were also not significant (Table 1). The ISOL fish results (time in ROI = 22.67 ± 3.17%, mean ± SEM, n = 10) matched what would be expected from a uniform distribution in the arena, with the fish showing no particular preference for the ROI and spending on average 25% of the time in 25% (ROI) of the total area (One-sample *t*-test, *t*_9_ = –0.73, *P* = 0.48). In the BNIC treatment, three (time in ROI = 84.39 ± 4.48%, n = 3) out of the 12 tested fish showed a strong proximity towards the stimuli, which differed from the other nine fish (time in ROI = 27.03 ± 4.46%; n = 9) suggesting a possible bimodality of a subset of the sampled population. To measure the focal fish’s directionality, we analysed individual and group preferred orientations, and also directional focus (see Methods for details) towards the stimulus fish. Circular scatter plots of the individual mean resultant vector’s orientations and directional focus (see Methods), revealed different distribution patterns for each treatment (Fig. 2b,d). We observed that BIC fishes’ mean orientations, strongly clustered around the fighting conspecifics tank’s direction (at 180 degrees). BNIC fish also oriented predominately towards the stimulus direction although scattered as well around other directions, whereas ISOL fish showed a dispersed distribution along different directions. Correspondingly, determination of the group mean resultant vector for each treatment (see Methods, Fig. 2b) revealed that all group mean resultant vectors were oriented towards the stimuli at 180°[*α*_*g*_ (BIC) = 182.59°, 95% C.I. = 158.43° – 191.91°, *R*_*g*_ = 0.25, n = 11; *α*_*g*_ (BNIC) = 179.07°, *R*_*g*_ = 0.14, n = 12; *α*_*g*_ (ISOL) = 186.21°, *R*_*g*_ = 0.042, n = 10], with the corresponding mean vector’ lengths *R*_*g*_, a measure of directional focus (see Methods for details), showing a higher value for the BIC treatment. Likewise, the individual fish vector’s length projection (*R proj*) onto the stimulus direction and the corresponding group mean projection (*R*_*g*_ *proj*) (see Methods and Fig. 2e), were significantly higher for BIC fish than ISOL fish, whereas there were no differences between BNIC and ISOL fish (see Table 1). The differences between BIC and BNIC fish were also not significant (Table 1). Reassuringly, circular uniformity analysis confirmed that only the BIC fish showed a significant directional focus towards the stimulus, with their individual mean orientations’ distribution (Fig. 2b) deviating significantly from uniformity and clustering around the corresponding group mean direction[Moore’s Test, (BIC): *P *< 0.001; (BNIC): *P *> 0.1; (ISOL): *P *> 0.1].

Next, we measured the bystanders’ locomotor activity (measured by the total distance covered in the arena and mean speed in the ROI) and stress levels (measured by whole-body cortisol concentration) to make sure that the observed differences in attentional measures across treatments where not mediated by any of these variables. The total distance covered (Fig. 2f) in the arena and mean speed in the ROI (Fig. 2g) did not differ significantly across treatments (Table 1). Post-test whole-body cortisol levels were also not significantly different across treatments (Fig. 2h, Table 1).

Finally, we compared the temporal dynamics of the BIC fishes’ mean time in ROI and *R*_*g*_ *proj*, with the ISOL reference group. We observed that both mean values were sustained throughout the 30 minutes test (Fig. 3a,b) and that the two parameters strongly correlated with each other (Spearman correlation: *r*_s_ = 0.70, *P *< 0.001; Fig. 3c).

Together, the results presented here show that zebrafish were strongly drawn towards interactions between conspecifics. However, it is not possible to control either the behaviour of an individual fish or the interaction dynamics between fish. Thus, in order to standardize the interacting conspecifics stimulus presented to the focal fish, we decided to test if video playbacks (previously used in zebrafish in other behavioural tasks^26^,^37^) could also be used to test attention to these stimuli.

### Video playbacks of social stimuli confirm that zebrafish attention is tuned to social interactions among third parties

**Figure 4:**
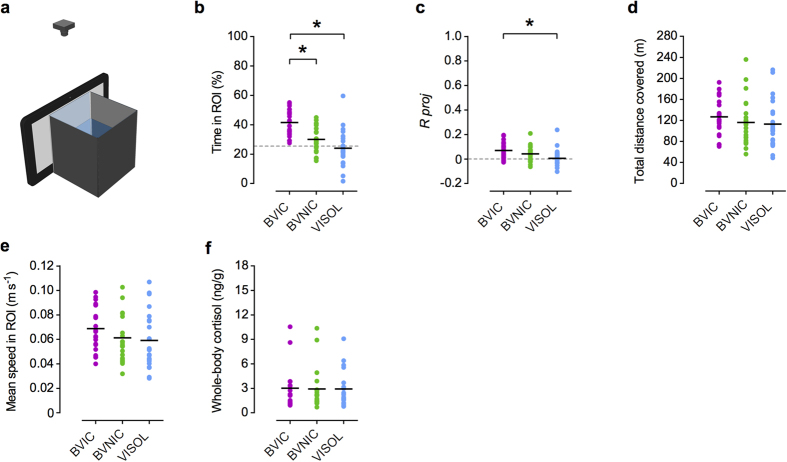
Bystanders to video playbacks’ behavioural and hormonal results. (**a**) Video playback setup: a tablet display replaces the demonstrator tank in the original experimental setup (see Methods). (**b**) Scatter plot (n = 23 / treatment) of the individual (coloured dots) and mean (black lines) percentage of time spent in the ROI for each treatment[bystander to video of fighting conspecifics (BVIC) - dark magenta; bystander to video of non-interacting conspecifics (BVNIC) - green; observing video of empty tank (VISOL) - light blue]. Dashed grey line represents the value expected from a random distribution in the arena (25%). (**c**) Scatter plot of the individual (coloured dots) resultant vectors’ lengths *R* projected (*R proj*) onto the stimulus direction (180˚) and corresponding group mean value *R*_*g*_ *proj* (black lines) for each treatment. Positive values indicate directional focus towards the stimulus; zero indicates no directionality (dashed grey line); negative values indicate directional focus opposite to the stimulus. (**d,e,f**) Scatter plots of the individual (coloured dots) and mean values (black lines) of the focal fishes’ total distance covered in the arena, mean speed in ROI and whole-body cortisol levels, for each treatment.

In a second experiment, we modified our experimental setup by replacing the demonstrator tanks with a video display (see Fig. 4a and Methods). New focal fish were tested for 30 minutes using video projections as stimuli, in one of three treatments: (1) bystander to a video of fighting male conspecifics (BVIC); (2) bystander to a video of non-interacting male conspecifics (BVNIC); and (3) observing a video of an empty tank (VISOL).

Similarly to the results obtained using real stimuli, bystander focal fish spent more time in close proximity to the stimulus (Fig. 4b) and showed higher directionality when presented with a video of fighting conspecifics. Specifically, BVIC fish spent significantly more time in the ROI than either BVNIC or VISOL fish (see Fig.4b, Table 2). Moreover, there were no significant differences between BVNIC and VISOL fish (Table 2). Both BVIC and BVNIC group mean vectors were oriented towards the stimulus at 180°[*α*_*g*_ (BVIC) = 191.38°, 95% C.I. = 178.13° – 216.45°, *R*_*g*_ = 0.071, n = 23; *α*_*g*_(BVNIC) = 185.71°, C.I. = 167.64° – 227.39°, *R*_*g*_ = 0.043, n = 23; *α*_*g*_(VISOL) = 242.53°, *R*_*g*_ = 0.013, n = 23]. Although all group vector lengths *R*_*g*_ showed values proximate to zero (no focus), the group mean vector length’s projection (*R*_*g*_ *proj*) onto the stimulus direction (Fig. 4c), was significantly higher for the BVIC treatment than the BVNIC treatment, when compared to VISOL (Table 2). However, there was no significant difference in *R*_*g*_ *proj* between BVIC and BVNIC fish (Table 2). Also, the distribution of the individual fish’s mean orientations for BVIC and BVNIC treatments deviated significantly from uniformity towards the corresponding group mean direction (Moore’s test: *P *< 0.001 for both cases; see Supplementary Fig. 1).

Similarly to what happened in the first experiment neither differences in locomotor or stress levels across the three treatments, explains the differences in attentional parameters between treatments. Analyses of the total distance covered and mean speed in the ROI did not reveal significant differences between treatments (Table 2; Fig. 4d,e). Whole-body cortisol levels also did not show significant differences between treatments (Table 2; Fig. 4f).

Overall these results confirm those obtained with real conspecifics in the first experiment, hence suggesting that zebrafish respond to video playbacks of conspecific stimuli. Importantly, in the video playback experiment the differences between BVIC and BVNIC in the time spent in proximity to the conspecific stimuli became significant, which supports the hypothesis that zebrafish attention is particularly tuned to social interactions. However, an alternative hypothesis equally supported by these results is that zebrafish may be more attracted to interacting than to non-interacting conspecifics, simply because the amount of movement (e.g. average speed of stimuli fish) in social interactions is higher than in non-interacting dyads.

### Zebrafish attention towards social interactions is not merely associated with levels of activity of the stimuli

**Figure 5:**
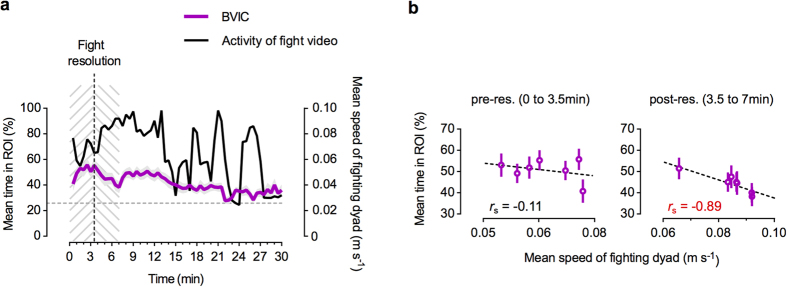
Video fight activity vs. bystanders’ proximity to the screen. (**a**) Temporal dynamics of the mean speed of the fighting dyad (black curve) in the video and the BVIC fishes’ mean time spent in the ROI (dark magenta curve), measured in 30 seconds bins, throughout the 30 minutes test. Grey shadow represents the standard error (SEM); dashed grey horizontal line - value expected from a random distribution in the arena (25%); dashed black vertical line - video fight resolution time point (at 3.5 minutes); dashed grey areas - pre-resolution (0 to 3.5 min) and post-resolution (3.5 to 7 min) time intervals analysed. (**b**) Scatter plots of the BVIC fishes’ mean time spent in the ROI as function of the mean speed of the video’s fighting dyad (video activity), before (0 min to 3.5 min) and after (3.5 min to 7 min) the fight resolution point. Open circles and error bars represent the sampled (in 30 seconds bins) mean ± SEM points. The Spearman correlation coefficient *r*_s_ is shown in red when significant (*P *< 0.05). Dashed lines indicate the regression line for easier visualization of trends.

In order to distinguish between the hypotheses proposed above, we tested if the time spent in close proximity to the screen was correlated with a measure of the stimulus movement on screen. For this purpose we determined the mean speed of the fighting dyad in the video (see Fig. 5a) in 30 seconds bins, as a measure of activity on screen throughout the 30 minutes test. The fighters’ activity profile was heterogeneous, revealing a steep increase in their mean speed after the fight resolution (i.e. time at which a dominant and a subordinate emerged in the fight), resulting from high speed chasing of the subordinate by the dominant, followed by alternating periods of inactivity and chasing bouts. Next, we performed a correlation analysis (Fig. 5b) before (0 min to 3.5 min) and after (3.5 min to 7 min) the fight’s resolution point, which occurred at 3.5 minutes into the video, using 30 seconds bins as samples units, in order to compare the BVIC fishes’ mean time in the ROI with the activity in the video. The results showed no correlation between the mean percentage of time in ROI and mean speed of the fighting dyad before the fight resolution (Spearman correlation: *r*_s_ = −0.11, *P* = 0.84), and showed a strong negative correlation after the resolution (Spearman correlation: *r*_s_ = −0.89, *P* = 0.012). Thus, the mean speed of the fighting dyad on screen during the time period around the fight resolution, was either not correlated or negatively correlated with the bystanders’ mean time spent in the ROI, suggesting that social features rather than conspicuousness of the conspecific dyad drive zebrafish attention towards social interactions. We further investigated this hypothesis experimentally by editing the video clip of the fighting dyad used for the video playbacks.

### Social features drive zebrafish attention towards social interactions

In a third experiment we tested if it is the type of movement present in the video images or specific form features present in the social interaction, that drive zebrafish attention. For this purpose, we also included a treatment: bystander to a video of fighting dots (BVID), where we manipulated the fighting conspecifics video by replacing both fish by dots (see Methods and Supplementary Video 2), while maintaining the original movement. We predicted that if it is the structure of movement of the fighting dyad that drives bystanders’ attention, BVIC and BVID should exhibit similar levels of time spent in close proximity to the stimulus. On the other hand, if specific form features present in the social interaction are relevant for attracting zebrafish attention, BVIC should display higher levels than BVID.

**Figure 6:**
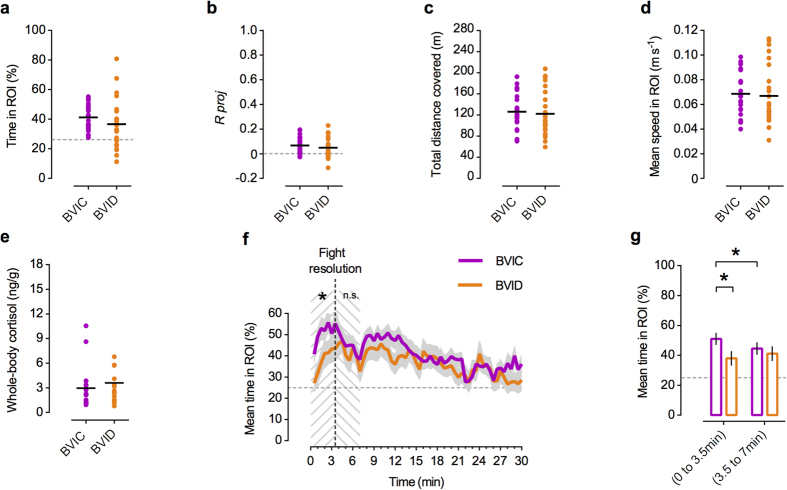
Bystanders to fighting conspecifics versus bystanders to fighting dots. (**a–e**) Scatter plots (n = 23 to 24 / treatment) of individual (coloured dots) and mean (black lines): time spent in the ROI; resultant vector’s length *R* projected (*R proj*) onto 180˚; total distance covered; mean speed in ROI; and whole-body cortisol levels (n = 18 to 19 / treatment), for the bystander to video of fighting conspecifics (BVIC - dark magenta) and bystander to video of fighting dots (BVID - orange) treatments. Grey dashed line represents in (**a**) the value expected from a random distribution in the arena (25%); and in (**b**) no directionality (*R proj* = 0). (**f**) Temporal dynamics’ comparison of the mean time spent in ROI, between BVIC (dark magenta) and BVID (orange) treatments. Grey shadows represent the standard error (SEM); dashed grey horizontal line – value expected from a random distribution in the arena (25%); dashed black vertical line - video fight resolution time point (at 3.5 minutes); dashed grey area – pre-resolution (0 to 3.5 min) and post-resolution (3.5 to 7 min) analysed time intervals. (**g**) Bars plot of mean ± SEM comparison between BVIC and BVID treatments, before and after the fight resolution event, in the previously defined time period (* *P *< 0.05).

No significance differences in the mean time spent in the ROI were detected between BVID and BVIC (Table 2) for the 30 minutes analysis, although BVID fish revealed twice the dispersion of BVIC (Fig. 6a). The BVID group resultant mean vector also oriented towards the stimulus[*α*_*g*_ (BVID) = 176.75°, 95% C.I. = 219.87°–106.32°, *R*_*g*_ = 0.051, n = 24), with the distribution of the individual fish’s mean orientations deviating significantly (Moore’s test, *P *< 0.005) from uniformity (Supplementary Fig. 1). The value of its *R*_*g*_ *proj* onto the stimulus direction was also low and not different from BVIC (Table 2; Fig. 6b). Analysis of the total distance covered and mean speed in ROI, did not reveal significant differences to the BVIC treatment (Table 2; Fig. 6c,d). Whole-body cortisol levels were also not significantly different (Table 2; Fig. 6e).

However, because we had shown above that the activity of the stimulus dyad was heterogeneous along the 30 minutes of the interaction, with a steep increase after the fight resolution point, we further analysed the temporal dynamics of both BVIC and BVID fishes’ mean time spent in the ROI throughout the test (see Fig. 6f and Methods). Particularly, we further analysed the adjacent time intervals before (0 min to 3.5 min) and after (3.5 min to 7 min) the fight resolution point. Accordingly, we compared BVIC and BVID using two equal time bins of 3.5 minutes each, before and after the event (Fig. 6f,g). The results revealed a significant difference between the two treatments before the fight was resolved but not after[Repeated Measures ANOVA, interaction: *F*_1,45_ = 5.23, *P* = 0.027; Contrasts (BVIC-BVID pre-resolution): *t* = 2.06, *P* = 0.045; Contrasts (BVIC-BVID post-resolution): *t* = 0.53, *P* = 0.6]. Results also showed there was a significant decrease (Fig. 6f,g) in the BVIC fishes’ mean time spent in ROI[Contrasts (pre vs. post-resolution): *t* = 2.12, *P* = 0.039] after the fight was resolved, which did not happen in the BVID treatment[Contrasts (pre vs. post-resolution): *t* = 1.10, *P* = 0.27].

Together these results indicate that during the pre-resolution phase of the fight, in which interacting fish are signalling to each other their competitive ability using ritualized displays, bystanders’ attention is not explained by the activity levels or structure of movement of the stimulus fish alone, suggesting that attention is tuned to relevant form features present in signalling interactions.

## Discussion

The results presented here show, for the first time that the attention of a highly social species is tuned to interactions between conspecifics. This conclusion is based on the fact that zebrafish males are more attentive towards interacting than towards non-interacting conspecifics, together with the fact that this interest is not due to heightened activity levels of the interacting conspecifics making them more conspicuous to bystanders. Therefore, zebrafish bystanders’ attention seems to be attracted by specific form or movement features present in social interactions between conspecifics.

Interestingly, the features that drive bystanders attention towards social interactions vary with the interaction dynamics. In the initial phase of the agonistic interaction, when opponents mutually assess each other’s competitive ability^38^, bystanders’ attention towards fighting conspecifics is higher than towards fighting dots, and with a much smaller variability, indicating that form features play a key role at this stage. This is not surprising since the information being exchanged by the opponents at this assessment phase is mainly based on the display of species-specific stereotyped action patterns (e.g. lateral displays), which imply changes in form features rather than changes in the whole fish movement. Therefore, in order to extract relevant information on the relative competitive ability of observed conspecifics, bystanders should focus their attention on form features during this signalling phase of the interaction. Potentially relevant form features are the shape of a conspecific’s body contour or the typical striped colouration pattern. Both are good candidates to drive attention in zebrafish, since during agonistic interactions, lateral displays imply changes in body contour (i.e. spreaded fins), together with changes in the intensity of body colouration also observed in aroused zebrafish^39^. Moreover, the striped colouration and other form features are known to play a key role in the social approach response towards conspecifics in zebrafish^30^,^40^. Classic ethology studies have demonstrated the role of such simple form features of complex stimulus (aka sign stimuli or releasers) in triggering the expression of species-specific behavioural action patterns across different species (e.g. attack response of breeding male sticklebacks, *Gasterosteus aculeatus*, towards dummies with red bellies^41^,^42^,^43^). Additionally it has been shown that zebrafish can integrate form and motion (aka feature binding) in a cohesive perceptual representation^44^. Therefore, the strong tendency to face the opponent in the assessment phase, which is absent in the post-resolution chasing phase, may provide specific information to eavesdroppers about the fight status, which is lost when the form features are replaced by dots. One can speculate that tuning of attention towards sign stimuli must be also part of the cognitive process that leads to an effective behavioural response. Since sign stimuli trigger the expression of adaptive behaviours in conspecifics, such cognitive processes, including selective attention, must have co-evolved with the relevant form feature. Therefore, it is expected that search images (i.e. mental images of relevant features that enhance their detectability), which have been described in the context of foraging search behaviour^45^, may also be present in the social domain. Search images for conspecific form or movement features would be an effective way for social animals to enhance the acquisition of social information in detriment of other environmental information, similarly to the limited attention constraint that has been demonstrated for prey search images in visual predators^46^. Like foraging search images that can be updated based on past experience of relative abundance for different food items^47^, social search images may also be updated by experience or context. Future studies are needed to explore these possibilities.

After the fight resolution, when a clear dominant-subordinate role has been established between the interacting fish, bystanders’ attention towards fighting conspecifics and towards fighting dots becomes similar, indicating that at this stage of the fight, movement features, rather than form features, are relevant to explain attention levels. Interestingly, proximity levels immediately after the resolution phase were not positively correlated with movement on the screen, as measured by the average speed of the fighting conspecifics, indicating that attention levels, although being driven by movement features, are not driven by the conspicuousness of the visual stimulus. Therefore, other movement parameters are needed to explain bystanders’ attention after the fight resolution. At this stage the overall behaviour of the interacting agents (either fish or dots) is dominated by movement components (dominants chase and attack; subordinates flee), whereas form components (e.g. lateral displays) are virtually absent. These movement features are common both to fighting fish and fighting dots, which may explain the lack of difference in the response to these two stimuli, observed at this stage of the fight. It is known that both humans and non-human animals, including fish, are tuned to attend to biological motion^48^,^49^,^50^,^51^, characterized by intrinsic accelerations and changes in direction of the behavioural agent without the action of an external cause (e.g. change of direction due to hitting an obstacle). These animacy movement features are present both in the agonistic action patterns expressed during the display phase and during chasing, and can therefore play a key role in attracting the attention of bystanders. Research on pre-verbal human infants has shown that they are more attentive towards the biological motion of two behavioural agents when social contingency is present (i.e. chasing), than when they move independently from each other^52^,^53^. Moreover, some characteristics of chasing enhance its perceptual value, such as role reversal between the two agents (i.e. chaser and evader switching roles), “heat-seeking” chases (i.e. chaser taking the shortest path to the evader), and coherence of the orientation of the chaser according to its path of travel^54^,^55^,^56^. The tuning of attention to chasing, both in zebrafish and in humans, might represent a conserved bias in attentional processes towards a fitness relevant cue in the environment. Indeed, the outcome of a chase typically has fitness consequences, whether these being for prey to successfully escape a predator, for a predator to successfully capture its prey, or for a subordinate individual to avoid being harmed by a dominant. Finally, it should be mentioned that despite the fact that zebrafish can perform feature binding, and therefore might integrate different features of chasing to extract meaning in terms of dominance relationships, in humans attention to chasing seems to be based on its movement features, in particular acceleration of the agents, rather than on the configuration of its features^53^.

From a functional perspective the tuning of zebrafish attention to social interactions can be seen as an adaptive specialization to group living, since it allows the individual to eavesdrop on social interactions between third parties. Social eavesdropping on aggressive interactions, where bystanders use the collected information to infer dominance relationships and therefore to adjust their behaviour in subsequent interactions with the observed conspecifics, has been documented for other fish species^2^,^57^. Future work should test the subsequent use of eavesdropped information in zebrafish.

## Methods

### Animals and Housing

Adult male wild-type (AB) zebrafish (*Danio rerio*), 11 months old, bred and held at Instituto Gulbenkian de Ciência (IGC, Oeiras, Portugal) were used. All fish were kept in mixed sex groups in environmentally enriched stock tanks (gravel substrate, artificial plants and rocks), with water temperature at 28 ^°^C and a 14L:10D photoperiod. Water was filtered and monitored for nitrites (<0.2 ppm), nitrates (<50 ppm) and ammonia (0.01–0.1 ppm). Conductivity and pH were maintained at 700 μSm and 7.5 respectively. Fish were fed twice a day with commercial food flakes in the morning and with freshly hatched *Artemia salina* in the afternoon, except on the day of the experiments. All experiments were performed in accordance with relevant guidelines and regulations, reviewed by the Instituto Gulbenkian de Ciência Ethics Committee, and approved by the competent Portuguese authority (Direcção Geral de Alimentação e Veterinária permit 008955).

### Behavioural setup

The experimental setup (Fig. 1a,b) consisted of three side-by-side test tanks (13 × 13 × 17 cm each) and three demonstrator tanks (15 × 15 × 17 cm each), one for each experimental treatment. The observation glass side of each test tank was positioned head-to-head to the end glass side of a demonstrator tank. A one-way mirror was placed in-between to prevent interactions between demonstrators and focal fish (bystanders), allowing each bystander full view of the demonstrator fish without itself being seen. A fluorescent light was placed over the demonstrator tanks, creating differential lighting required for the mirror effect. Each test tank had black opaque walls to enhance this effect, with the exception of the transparent glass observation side. This also avoided interference of spurious external visual cues. Each demonstrator tank had white opaque walls, except for the transparent glass end side. All tanks were filled up to a 9 cm water depth. Three B&W mini surveillance cameras (Henelec 300B) with infrared sensitivity (IRs) were placed above each test tank and connected to a laptop (HP Pavilion g6). This allowed a top view video recording of the focal fish and demonstrator fish simultaneously. The setup was placed over an infrared LED (850 nm) custom built lightbox, to increase contrast between the background of the test tanks and the focal fish when video recording, without interfering with their vision, as IR light falls outside zebrafish wavelength sensitivity^58^. This increased image quality and optimized subsequent video tracking of the fishes’ behaviour. A black curtain separated the setup from the rest of the behavioural room during the experiment and no person was allowed inside during the testing period.

### Experimental procedures

A total of 39 focal naïve male zebrafish were used (13 per treatment). Each fish was subjected to a single test corresponding to one of three treatments (Fig. 1c): bystander to male fighting conspecifics (BIC), bystander to non-interacting conspecifics (BNIC), and socially isolated (ISOL). The behavioural setup allowed testing three different bystanders per day. On the day prior to the test, three fish of similar size were randomly removed from the stock tanks and isolated in each test tank overnight. This produced an isolation baseline effect and allowed for setup acclimatization. The order of the treatments attributed to each tank was randomized for each session. To prepare the BIC and BNIC demonstrators, two pairs of unfamiliar zebrafish matched in size, where placed in the corresponding demonstrator tanks. A removable white opaque partition was placed between each pair overnight, allowing chemical but no visual communication. The ISOL treatment was prepared by keeping a demonstrator tank empty, with an opaque partition also placed in the middle to match the other tanks. Removable opaque partitions were additionally placed between each test tank and the one-way mirror, to prevent visual contact between demonstrators and bystanders during the isolation period. The demonstrators were allowed to habituate to the one-way mirror reflection overnight. This avoided interactions with the mirror during the tests. On the following day, at the beginning of each test, the opaque partition that visually separated each test tank from the corresponding demonstrator tank was removed. Each focal fish could then visually observe the corresponding demonstrator tank for 30 minutes. For the BIC treatment, the middle opaque partition separating the demonstrator dyad was also removed simultaneously, prompting demonstrators to fight. For the BNIC treatment, the middle partition remained in place, preventing the two demonstrators to interact. For the ISOL treatment, the middle partition also remained in place. All focal fish behaviours were video recorded for posterior offline behavioural tracking and analysis. Immediately after the test, each focal fish was euthanized with an overdose of tricaine solution (MS222, Pharmaq; 500–1000 mg/L) and sectioning of the spinal cord. Gender was confirmed by dissection of the gonads. Body samples were stored at −80 ^°^C for posterior whole-body hormonal analysis.

### Behavioural tracking

All focal fish behaviours were tracked from a top-down view perspective, using a custom made tracking software (see https://github.com/joseaccruz/fishtracker) developed in Python (python^TM^). For each behavioural video, a 2D region (arena) was defined for tracking (Fig. 1b). The arena’s position and size took into account the camera’s perspective distortion caused by the water depth and comprised the inner area of the bystander tank (12 × 12 cm) including the stimulus observation side, where the lighting contrast between the white background and the fish was high. It excluded the black outer walls sidelines where contrast was low. The fish were tracked at a 29 fps rate. The tracking software determined and extracted into data files, the pixel coordinates of the head, centroid, and tail for each frame (see Fig. 1d and Supplementary Video 1). This allowed determination of position and orientation (Fig. 1e) of the fish every 1/29 s. It also identified and counted all frames in which the fish was not detected. This only occurred at surface level, alongside the tank’s black outer walls (on average 4% of the total time). After tracking, the head, centroid, and tail coordinates were projected over the video (see Fig. 2a) allowing the manual inspection of the tracking quality and an easy early detection of possible tracking errors. 

### Behavioural data analysis

All tracked data files were imported to MATLAB (MathWorks ®) and behavioural parameters were determined using a custom-made script. A region of interest (ROI) with 12 × 3 cm (25% of the tank) corresponding to the width of the tank and the mean body length of an adult zebrafish, was defined in the area of the arena closest to the observation glass (Fig. 1c). The focal fish was considered in the ROI when its centroid point was inside that region. The following behavioural parameters were determined and analysed at an individual and group level for each treatment: total time spent at each position in the arena, total time at each direction, percentage of time spent in the ROI, mean speed in the ROI, total distance covered in the arena, mean preferred orientation in the arena and directional focus. The determination of the total distance covered by each fish took into account an estimation of the distance covered when the fish could not be tracked, by considering it proportional to the total distance covered when detected. To determine the mean resultant vector for the fish, each orientation taken during the 30 minutes test was first transformed to a unit vector 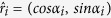. Where *α*_*i*_ was the angle formed by the fish’s centroid-to-head axis direction relative to the horizontal axis in each frame. The mean resultant vector was thus defined as the mean of all 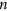 frames’ unit vectors taken by the fish during the testing period and calculated by 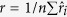. The mean resultant vector’s length 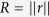, a measure of directional focus and inversely related to the angular standard deviation, was calculated by the mean vector’s norm and ranged from 0 to 1. The closer 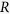 (Fig. 1e) is to one, the more concentrated the *n* orientations are around the mean direction. The projection of 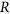 onto the stimulus tank’s direction (180°) was determined by 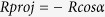, where *α* (Fig. 1e) is the angle formed by the mean resultant vector *r*. This allowed measurement of the mean directional focus of each fish relative to the stimulus direction, using a linear scale ranging from 1 to −1. Positive values indicate directionality towards the stimulus, negative values away from it and null values no directionality. For each treatment, the group mean resultant vector 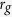, correspondent mean angle 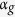 and length 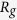, was determined by the grand mean of all focal fishes’ mean resultant vectors 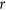, weighted by their individual lengths *R*.

The temporal dynamics and correlation of the BIC vs. ISOL treatments for the mean time spent in ROI and 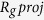, was calculated in 30 seconds bins (Fig. 3).

### Video playbacks-experimental setup and procedures

The original experimental setup was adapted by replacing the demonstrator tanks (Fig. 4a) with a 10-inch, 1024 × 768 LCD tablet, positioned adjoining the end glass side of a removable bystander tank. A camera was placed above the tank for a top-down view video recording and later tracking of the focal fish. The same lighting conditions were maintained to match the previous experimental settings. In this experiment, the number of bystander focal fish was increased to 23 per treatment. Each focal fish was subjected to a single 30 minutes test corresponding to one of four new treatments: (1) bystander to a video of fighting conspecifics (BVIC) comprising a pre-resolution, resolution, and post-resolution stage^31^; (2) bystander to a video of fighting dots (BVID), where the original fight video was manipulated by replacing the fighting fish by circles (dots), while maintaining the same original movements (see Supplementary Video 2); (3) bystander to a video of non-interacting conspecifics (BVNIC); and (4) observing a video of an empty tank (VISOL), as control for the stimuli and any possible effects of the screen itself. Each video presented was previously recorded with a digital video camera at a 25 fps and 720 × 576 pixel resolution, using the same conditions and settings of the previous experiment. The videos were displayed on the tablet using real size images.

On the day prior to the test, fish of similar size were randomly removed from the stock tanks and isolated in each bystander test tank overnight, next to the experimental setup. This produced an isolation baseline effect and allowed for setup lighting acclimatization. Removable white opaque partitions were placed on the observation glass side of each test tank to prevent visual contact with the outside. On the following day, prior to the beginning of each test, a test tank with an isolated focal fish inside was placed in the setup (with the opaque partition still in place), positioned in front of the tablet screen and allowed to habituate for 30 min. At the beginning of the test, the video started playing on the screen and the opaque partition was immediately removed. Each focal fish could then visually observe a video for 30 min. The order of the video treatments was randomized for each session. All focal fishes’ behaviours were video recorded for posterior offline behavioural tracking and analysis. Immediately after the test, each focal fish was euthanized. All samples were stored at −80 ^°^C for posterior analysis.

### Manipulation and activity analysis of the fighting conspecifics video

Replacement of the fighting fish by dots was achieved firstly by tracking and extracting both fighters’ centroid coordinates, size, colour and contrast for each frame, using a custom-made tracking software. Two circles with the mean area, colour and contrast of the original fish were then placed at the corresponding centroid positions, over the fish-subtracted background images of the tank. This allowed exact replication of the fighters’ movement, while eliminating their form features (see Supplementary Movie 2). As a measure of activity on screen throughout the 30 min video fight, the mean speed of the fighting dyad was calculated in 30 s bins (Fig. 5a) using the fighters’ tracked data. This allowed profiling the temporal dynamics of the fight’s level of activity and posterior correlation analysis with the bystanders’ mean time spent in the ROI, when observing the video fight.

### Hormonal analysis

Cortisol whole-body (WB) levels were measured for each focal fish. For the hormone extraction, the collected WB samples, kept at −80 °C, were first measured in body weight and length for normalization purposes. Each sample was partially thawed, weighed and dissected on ice into smaller parts for efficient homogenization. 500 μl of EIA Buffer (from Cayman EIA kit) were added and vortexed for 3 s. The samples were then transferred to extraction glass tubes and homogenized using a mechanical homogenizer (IKA Labortechnik) for 30 s on ice. The homogenization rotor blade was washed with additional 500 μl of ice-cold EIA buffer and collected in the glass tube containing the homogenate. Samples were sonicated for 30 s on ice, added 3 ml of diethyl ether, vortexed, stirred for 10 min in the orbital shaker and then centrifuged at 2000 rpm (4 °C) for 15 min. Following centrifugation, samples were frozen at −80 °C for 15 min and the organic layer (containing the hormones) was removed from each sample and placed in a separate test tube. Ether was evaporated with a speed vacuum centrifuge (Speedvac Savant SC 1101) equipped with a cryotrap. Samples were reconstituted in 1 ml of EIA buffer after evaporation and kept at −20 °C until analysis. Cortisol levels were assayed using enzyme immunoassay (EIA) kits from Cayman Chemical Company (#500360) following the manufacturer’s instructions. In the cases where samples were too concentrated, dilutions were performed and measurements repeated. For the first experiment, the intra-assay coefficient of variation was 3.20% and inter-assay coefficient of variation was 8.79%. For the video experiment, the intra-assay coefficient of variation was 5.10% and inter-assay coefficient of variation was 2.80%.

### Statistics

Behavioural and hormonal results were represented as mean ± SEM unless stated otherwise. Statistical significance was considered for *P *< 0.05. For the behavioural parameters’ comparisons between treatments, one-way ANOVAs were performed when normality and homogeneity of variances (Levene’s test) was verified, followed by post-hoc Tukey HSD tests or contrasts for specific planned comparisons. When normality was verified but not homogeneity of variances, Welch’s ANOVAs were used, followed by Games-Howell post-hoc tests. When normality was not verified, non-parametric Kruskall-Wallis ANOVAs were used. Cortisol concentrations were first ln transformed to meet the assumption of a normal distribution. Deviation from uniformity of the fishes’ individual mean orientations distribution was tested using the non-parametric Moore’s Modified Rayleigh test, for each treatment. The angles of the group mean resultant vectors were represented as mean, 95% C.I. when directionality was significant. Correlations were performed using a non-parametric Spearman rank correlation. All analyses were performed using MATLAB R2012b (MathWorks) with the CircStat toolbox^59^, STATISTICA 12 (Statsoft^Inc^), SPSS Statistics 22 (IBM) and Oriana 4 (Kovach Computing Services).

## Additional Information

**How to cite this article**: Abril-de-Abreu, R. *et al*. Social Eavesdropping in Zebrafish: Tuning of Attention to Social Interactions. *Sci. Rep*. **5**, 12678; doi: 10.1038/srep12678 (2015).

## Supplementary Material

Supplementary Information

Supplementary Video S1

Supplementary Video S2

## Figures and Tables

**Table 1:** Bystander experiment: behavioural and hormonal parameters results.

	Mean time in ROI (%)	*R*_*g*_*proj*(–1 to 1)	Total distance covered (m)	Mean speed in ROI (m s^–1^)	Whole-body cortisol (ng/g)
Mean ± SEM					
BIC (n = 11)	55.05 ± 7.22	0.25 ± 0.06	102.32 ± 5.25	0.04 ± 0.002	3.32 ± 1.40
BNIC (n = 12)	41.58 ± 8.20	0.14 ± 0.07	89.28 ± 3.85	0.04 ± 0.002	1.70 ± 0.53
ISOL (n = 10)	22.67 ± 3.17	0.04 ± 0.03	96.25 ± 7.54	0.04 ± 0.003	1.36 ± 0.26
ANOVA					
BIC × BNIC × ISOL	*F* (2, 30)^a^ = 9.31	*F* (2, 30)^a^ = 4.52	*F* (2, 30)^a^ = 1.86	*F* (2, 30)^a^ = 0.35	*H* (2, 33)^b^ = 0.19
	*P* = 0.002	*P* = 0.03	*P* = 0.18	*P* = 0.71	*P* = 0.91
Games-Howell					
BIC vs. ISOL	*P* = 0.003	*P* = 0.03			
BNIC vs. ISOL	*P* = 0.12	*P* = 0.40			
BIC vs. BNIC	*P* = 0.45	*P* = 0.49			

BIC – bystander to fighting conspecifics, BNIC – bystander to non-interacting conspecifics, ISOL – socially isolated.

^a^Welch’s ANOVA;

^b^Kruskal-Wallis non-parametric test.

**Table 2:** Bystander to video playbacks: behavioural and hormonal parameters results.

	Mean time in ROI (%)	*R*_*g*_*proj*(–1 to 1)	Total distance covered (m)	Mean speed in ROI (m s^–1^)	Whole-body cortisol (ng/g)
Mean ± SEM					
BVIC (n = 23)*	41.46 ± 1.82	0.07 ± 0.01	126.89 ± 7.24	0.07 ± 0.004	3.02 ± 0.61
BVID (n = 24)*	36.82 ± 3.37	0.05 ± 0.02	123.04 ± 8.74	0.07 ± 0.005	3.67 ± 1.02
BVNIC (n = 23)*	30.03 ± 1.77	0.04 ± 0.01	116.09 ± 9.18	0.06 ± 0.005	2.92 ± 0.56
VISOL (n = 23)*	24.00 ± 2.54	0.00 ± 0.02	112.83 ± 9.47	0.06 ± 0.005	2.93 ± 0.51
ANOVA					
BVIC × BVNIC × VISOL	*F* (2, 66) = 18.32	*F* (2, 66) = 4.73	*F* (2, 66) = 0.72	*F* (2, 66) = 1.32	*F* (2, 54) = 0.013
	*P* < 0.0001	*P* = 0.01	*P* = 0.49	*P* = 0.28	*P* = 0.99
Tukey HSD					
BVIC vs. VISOL	*P* < 0.0001	*P* = 0.009			
BVNIC vs. VISOL	*P* = 0.11	*P* = 0.19			
BVIC vs. BVNIC	*P* = 0.001	*P* = 0.40			
Planned comparisons					
BVIC vs. BVID	*t* = 1.32	*t* = 0.87	*t* = 0.31	*t* = 0.25	*t* = 0.24
	*P* = 0.19	*P* = 0.39	*P* = 0.75	*P* = 0.80	*P* = 0.80

BVIC – bystander to video of fighting conspecifics; BVID – bystander to video of fighting dots; BVNIC – bystander to video of non-interacting conspecifics; VISOL – observing video of an empty tank.

^*^Cortisol sample sizes: BVIC (n = 18), BVID (n = 19), BVNIC (n = 20), VISOL (n = 19).
