# Supplementary material for: Social Eavesdropping in Zebrafish: Tuning of Attention to Social Interactions
Source: Sci Rep. 2015 Aug 5;5:12678. doi: 10.1038/srep12678 (PMC4525141; doi:10.1038/srep12678)
Supplement: Supplementary Information [file srep12678-s1.pdf]

Social eavesdropping in zebrafish: Tuning of attention to social interactions

Rodrigo Abril-de-Abreu, José Cruz & Rui F. Oliveira

Supplementary Information

Supplementary Figure 1

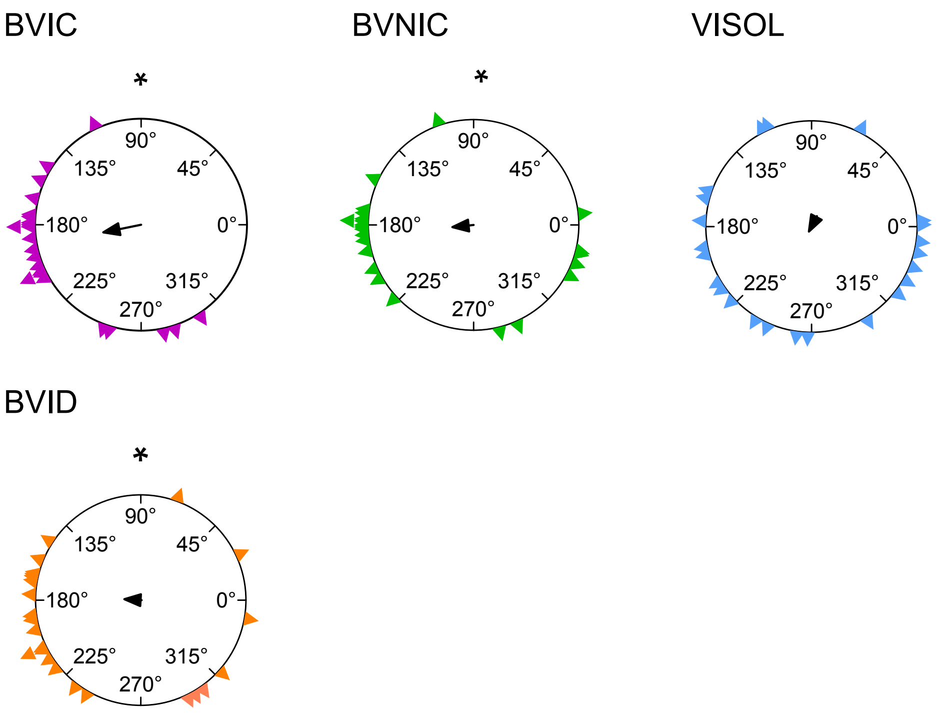

Supplementary Figure 1. Circular plots of the focal fishes' individual mean orientations for each treatment (BVIC – dark magenta triangles; BVID – orange triangles; BVNIC – green triangles; VISOL – light blue triangles) and the corresponding group mean resultant vector (black arrows). All distributions, except for the VISOL treatment, deviate significantly ($P < 0.05$) from a uniform distribution, clustering around the corresponding group mean resultant vector.

Supplementary Video 1. Video tracking samples of representative bystander focal fish from the following treatments: bystander to fighting conspecifics (BIC); bystander to non-interacting conspecifics (BNIC) and socially isolated (ISOL). A one-way mirror separates the stimulus tank (on the left) and the test tank with the focal fish (on the right). The tracking arena is represented by a magenta rectangle. Head, centroid and tail of the focal fish, are marked in red, green and blue, respectively.

Supplementary Video 2. Video playback samples of the 30 minutes video of fighting conspecifics and video of fighting dots, presented to BVIC (bystander to video of fighting conspecifics) and BVID (bystander to video of fighting dots) focal fish. Both video samples correspond to the same time interval. In the second video, the fighting fish were replaced by dots with the same area, mean color and original movement of the original fish.
